# Cross-cutting lessons from the Decision-Maker Led Implementation Research initiative

**DOI:** 10.1186/s12961-021-00706-0

**Published:** 2021-08-11

**Authors:** Arielle Mancuso, Shahira Ahmed Malm, Alyssa Sharkey, A. S. M. Shahabuddin, Zubin Cyrus Shroff

**Affiliations:** 1 Alliance for Health Policy and Systems Research, WHO headquarters, Avenue Appia 20, 1211 Geneva, Switzerland; 2 Nutrition Section, United Nations Children’s Fund, UNICEF headquarters, New York, NY 10017 United States of America; 3 Implementation Research and Delivery Science Unit, Health Section, United Nations Children’s Fund, UNICEF headquarters, New York, NY 10017 United States of America

**Keywords:** Immunization, Vaccination, Implementation Research, Embedded Research, Decision-Maker Led Research, Africa, Asia

## Abstract

**Background:**

Almost 20 million children under one year of age did not receive basic vaccines in 2019, and most of these children lived in low- and middle-income countries. Implementation research has been recognized as an emerging area that is critical to strengthen the implementation of interventions proven to be effective. As a component of strengthening implementation, WHO has called for greater embedding of research within decision-making processes. One strategy to facilitate the embedding of research is to engage decision-makers as Principal Investigators of the research. Since 2015, the Alliance for Health Policy and Systems Research within the WHO and the United Nations Children’s Fund have supported decision-maker led research by partnering with Gavi, the Vaccine Alliance, in an initiative called "Decision-Maker Led Implementation Research". This synthesis paper describes the cross-cutting lessons from the initiative to further understand and develop future use of the decision-maker led strategy.

**Methods:**

This study used qualitative methods of data collection, including a document review and in-depth interviews with decision-makers and researchers engaged in the initiative. Document extraction and thematic content analysis were applied. The individual project was the unit of analysis and the results were summarized across projects.

**Results:**

Research teams from 11 of the 14 projects participated in this study, for an overall response rate of 78.6%. Most projects were carried out in countries in Africa and conducted at the sub-state or sub-district level. Seven enablers and five barriers to the process of conducting the studies or bringing about changes were identified. Key enablers were the relevance, acceptability, and integration of the research, while key barriers included unclear results, limited planning and support, and the limited role of a single study in informing changes to strengthen implementation.

**Conclusions:**

Decision-maker led research is a promising strategy to facilitate the embedding of research into decision-making processes and contribute to greater use of research to strengthen implementation of proven-effective interventions, such as immunization. We identified several lessons for consideration in the future design and use of the decision-maker led strategy.

**Supplementary Information:**

The online version contains supplementary material available at 10.1186/s12961-021-00706-0.

## Background

Immunization is recognized as one of the most effective and economical public health interventions, preventing between two and three million deaths each year [1, 2]. Despite its promise, almost 20 million children under the age of one did not receive basic vaccines in 2019 [2]. Most of these children lived in low- and middle-income countries (LMICs) where coverage of vaccines remains lower than in high-income settings [3, 4]. For example, coverage of diphtheria-tetanus-pertussis-containing vaccine was 21% lower in low-income countries compared to high-income countries in 2019 [4]. Disparities in coverage of vaccines also exist within countries, with rural areas, urban slums, poorer households, and indigenous communities often experiencing lower coverage [5, 6].

Implementation research has gained attention as an emerging area within the field of health policy and systems research (HPSR) that is critical to strengthen the implementation of proven-effective interventions toward the achievement of international health goals [7, 8]. Implementation research is defined as the “scientific inquiry into questions concerning implementation—the act of carrying an intention into effect, which in health research can be policies, programmes, or individual practices (collectively called interventions)” [9]. Since implementation research focusses on issues of implementation that occur within real-world contexts and systems, there is significant value in engaging the actors involved in implementation in efforts to understand and strengthen it.

Since 2012, WHO has called for the greater embedding of research within health policy and programme implementation and systems strengthening efforts as a means to move closer towards universal health coverage [10, 11]. Embedding is an innovative approach to HPSR in which the research is carried out as an integrated component of decision-making processes [12]. The embedded approach is relevant to implementation research as efforts to address challenges and strengthen implementation ultimately rely on the decisions and actions of decision-makers and stakeholders operating in these systems.

Several strategies and techniques to facilitate the embedding of research have been employed, one of which is to engage decision-makers as Principal Investigators of the research (decision-maker led) [12, 13]. Since 2015, the Alliance for Health Policy and Systems Research (AHPSR) within WHO and the United Nations Children’s Fund (UNICEF) have supported decision-maker led research by partnering with Gavi, the Vaccine Alliance (GAVI) in an initiative called “Decision-Maker Led Implementation Research” (DELIR). Through this initiative, a total of 14 decision-maker led implementation research projects were completed in 10 LMICs with the aim of strengthening the implementation of immunization interventions and improving coverage.

This paper describes a study to synthesize the cross-cutting lessons from the initiative to further understand and develop future use of the decision-maker led strategy. Our objectives are: (1) to describe and summarize the projects, methods, activities, use, and perceived changes resulting from the projects; and (2) to examine the experiences and perceptions of decision-maker led research by both decision-makers and researchers engaged in the projects to identify enablers and barriers to conducting the research and using the results to bring about changes.

## Methods

The study was qualitative and consisted of both a document review and in-depth interviews with decision-makers and researchers engaged in the DELIR initiative. The initiative and data collection methods for this study are described in more detail below.

### Decision-Maker Led Implementation Research initiative

A full description of the DELIR initiative, its rationale, and processes is included in the editorial for this journal series [14]. For the projects supported through the DELIR initiative, the Principal Investigator had to be a decision-maker directly involved in the implementation of an immunization intervention (decision-maker led) working in collaboration with a researcher who was affiliated with a local academic or research institution. An overview of the supported projects participating in this study is provided in Table 1.

**Table 1:** Project summary

Call	Country	Region	Intervention	Priority issue	Level of study
2015	Chad	Africa	Expanded Programme on Immunization	Demand-creation communication strategies	District
2015	Nigeria	Africa	Expanded Programme on Immunization	Vaccination and coverage	Substate (local government area)
2015	Uganda	Africa	Expanded Programme on Immunization	Programme management, monitoring and evaluation strategies	District
2015	Vietnam	Western Pacific	Immunization Policy	Health and immunization systems	Provincial
2016	Ethiopia	Africa	Expanded Programme on Immunization	Health and immunization systems	Regional
2016	Ethiopia	Africa	Expanded Programme on Immunization	Health and immunization systems	Subdistrict (subcity)
2016	India	South-East Asia	Expanded Programme on Immunization	Demand-creation communication strategies	District
2016	Nigeria	Africa	Expanded Programme on Immunization	Demand and vaccine hesitancy	Substate (local government area)
2016	Nigeria	Africa	Expanded Programme on Immunization	Barriers to immunization services in urban slums	Substate (urban slum)
2016	Pakistan	Eastern Mediterranean	Expanded Programme on Immunization	Barriers to immunization services in urban slums	Subdistrict (urban slum)
2016	Uganda	Africa	Expanded Programme on Immunization	Health and immunization systems	District

### Document review

We conducted a review of relevant documentation from the initiative including final protocols, progress reports, final reports, policy briefs, and other research outputs for the studies shared with AHPSR, UNICEF, and GAVI.

#### Final protocol

Teams completed and submitted protocols based on a template. Space was made in the background section for describing the context and health system, immunization intervention, current implementation barrier and systems failure, and the knowledge that was needed to strengthen implementation. Emphasis was also placed on describing the plans for knowledge use, dissemination, and stakeholder engagement in the protocol.

#### Progress report

Approximately one year after the start of their projects, each team submitted a progress report. These reports included a description of the team’s activities and the progress that had been made towards achieving their study goals and outcomes. Teams also identified any challenges they were experiencing in carrying out their project and their plans for the next period.

#### Final report

Teams submitted a final report at the end of the research. These described all activities carried out during the project as well as the results in relation to the study goals and objectives.

#### Research outputs

Teams developed additional research outputs such as manuscripts, policy briefs, and presentations that were submitted along with their final reports [15–22].

### In-depth interviews

Respondents for in-depth interviews were purposively selected based on their role in the project. All decision-makers serving as Principal Investigators and their researcher counterparts were approached through an email introducing them to the study and inviting them to participate (two per project totaling 28). If a selected team member was unable to participate, they were invited to refer another team member to be approached for an interview.

Interviews were conducted individually with participating decision-makers and researchers using Skype or Zoom by one of the authors (SM). SM had no prior relationship with the study teams. The interviews followed a semi-structured guide (Additional file 1: In-depth interview guide questions). Interviews were audio-recorded and directly transcribed for analysis. Notes were also taken during each interview. Each interview lasted approximately one hour. No repeat interviews were carried out and the transcripts were not returned to participants. Since the purpose of the interviews was to understand the diversity of experiences and perceptions, saturation was assessed at the time of data analysis to determine the pervasiveness of emerging themes.

### Data analysis

Project documentation and interview transcripts were uploaded to NVivo 12© (QSR International, Doncaster, Australia) for organization and management. The primary unit of analysis was the project. First, characteristics of the projects were extracted and summarized across the projects by one of the authors (AM) using a data extraction form. Then, thematic content analysis was applied by AM in consultation with authors SM and ZS [23–25]. For the content analysis, initial codes for enablers and barriers were first developed. Factors were considered enablers when respondents identified them as contributing positively to carrying out the study or bringing about change. Factors were considered barriers when respondents discussed them as negatively influencing the study or their ability to bring about change or identified them as an aspect that could have been improved in their study. If a factor was discussed both positively and negatively by respondents, the number of respondents was used to determine whether it was considered to be an enabler or barrier and these different views were noted. This was followed by an iterative process of reading, examination, reorganization, and consultation to produce a final list of enablers and barriers. The final list included those enablers and barriers that were most pervasive in the data or most significantly influenced the process and change from the projects. This approach allowed themes to emerge from the data that were then summarized and presented across the projects.

## Results

In this section, we first describe participation of the research teams in this study and provide an overview of the projects. We then identify the enablers and barriers to the process of conducting the studies and bringing about changes.

### Participation of the research teams

Research teams from 11 of the 14 projects agreed to participate in this study, for an overall response rate of 78.6%. A total of 17 interviews were conducted with seven decision-makers and 10 researchers. For six projects, both the decision-maker and researcher participated in an interview. Only the researcher participated in an interview for four projects and only the decision-maker for one.

### Project characteristics

The participating projects (*n* = 11) were conducted in seven countries in four WHO regions, mostly Africa (*n* = 8; 72.7%) (Table 1). The intervention (policy, programme, or service) for almost all projects was the Expanded Programme on Immunization (*n* = 10; 90.9%). The greatest number of projects focused on health and immunization systems as the priority issue from the call (*n* = 4; 36.4%). Most projects were at the substate/subdistrict level (ex. local government area, subcity, urban slum) (*n* = 5; 45.5%) followed by the state/district (*n* = 4; 36.4%).

Almost all projects were exploratory (*n* = 9; 81.8%) and only two were interventional (*n* = 2; 18.2%) (Table 2). Almost all projects employed a mixed methods study design (*n* = 8; 72.7%). The most frequent primary data collection methods were qualitative, including focus group discussions (*n* = 10; 90.9%), key informant interviews (*n* = 7; 63.6%), and in-depth interviews (*n* = 6; 54.5%). Coverage remained the most common implementation outcome (*n* = 7; 63.6%), but acceptability (*n* = 6; 54.5%), adoption (*n* = 5; 45.5%), and sustainability (*n* = 4; 36.4%) were also frequently assessed.

**Table 2:** Research methods

Country	Project title	Primary type	Study design	Data collection methods	Implementation outcomes
Chad	More responsive immunization services through tailoring for hard-to-reach populations in Chad	Interventional	Mixed methods	Focus group discussions; key informant interviews; secondary data; survey	Feasibility; sustainability; appropriateness; acceptability; adoption
Ethiopia	How can the use of data within the immunization programme be increased in order to improve data quality and ensure greater accountability?	Exploratory	Mixed methods	Key informant interviews; document review	Sustainability; adoption
Ethiopia	Lack of functional linkages and feedback mechanisms among different health facilities along with mobility of caregivers affect follow-up visits in utilizing the routine immunization services	Exploratory	Cohort	Secondary data; in-depth interviews; key informant interviews; focus group discussions	Coverage
India	Negative social media messages on vaccines: how can the resultant trust deficit between caregivers and health workers be overcome? A qualitative inquiry in Malappuram district of Kerala State in India	Exploratory	Qualitative	In-depth interviews; focus group discussions; document review	Coverage; appropriateness; acceptability
Nigeria	Increasing the utilization of immunization in Ogun State of Nigeria using participatory evaluation and action research	Interventional	Participatory action research	Survey; secondary data; in-depth interviews; focus group discussions	Coverage; acceptability; feasibility
Nigeria	Potential role of civil society organization engagement for increasing the demand for and uptake of immunization services in Odukpani Local Government Area of Cross River State of Nigeria	Exploratory	Mixed methods	Secondary data; survey; focus group discussions; in-depth interviews; key informant interviews	Coverage
Nigeria	Use of social actors to address contextual barriers for utilization of immunization services among caregivers of under-five children in urban slums of Yobe State, Nigeria in the context of the Boko Haram insurgency	Exploratory	Mixed methods	Key informant interviews; survey; focus group discussions	Coverage; adoption
Pakistan	Improving vaccine uptake in urban slums of Karachi, Pakistan—implementation research to explore and address supply- and demand-side barriers to routine immunization	Exploratory	Mixed methods	Survey; focus group discussions; in-depth interviews	Adoption; coverage
Uganda	Process evaluation of community health facility-based microplan development and implementation in two districts in Uganda	Exploratory	Mixed methods	Observation; document review; key informant interviews; focus group discussions	Fidelity; adoption; acceptability
Uganda	Evaluating the role of leadership in transitioning vertical into integrated and sustainable district health programmes—a case study of immunization in Luuka district, Uganda	Exploratory	Mixed methods	Document review; key informant interviews; focus group discussions; observation	Acceptability; sustainability
Vietnam	Governance of the immunization programme for children 0–23 months in Vietnam	Exploratory	Mixed methods	Document review; in-depth interviews; focus group discussions; survey	Sustainability; coverage; acceptability

For almost all of the projects, the aim was to identify facilitators and barriers to implementation (*n* = 10; 90.9%) (Table 3). Seven (*n* = 7; 63.6%) projects focussed on describing an immunization-related problem. Only four (*n* = 4; 36.4%) and two (*n* = 2; 18.2%) projects, respectively, sought to explore potential implementation strategies or assess or test an implementation strategy. The projects described multiple challenges related to the implementation of immunization interventions (*n* = 7; 63.6%) and identified both demand- and supply-side barriers (*n* = 6; 54.5%). A few implementation strategies were explored and tested by the projects, such as a tailored communication strategy and participatory action research.

**Table 3:** Study results

County	Implementation significance	Vaccine-related problem	Implementation facilitators/barriers	Implementation strategies
Chad	Identifying implementation facilitators/ barriers; assessing or testing implementation strategies	N/A	*Demand-side barriers*: mistrust of the expanded programme on immunization and polio vaccination services; health systems issues; concerns related to potential harm of vaccines	OneHealth strategy; tailored communication strategy
Ethiopia	Describing a vaccine-related problem; identifying implementation facilitators/barriers; exploring potential implementation strategies	Use of data in immunization programme	*Supply-side barriers*: awareness gaps; lack of motivating incentives; irregularity of supportive supervision; lack of community engagement in health report verification; poor technical capacity of health providers	Community engagement
Ethiopia	Describing a vaccine-related problem; identifying implementation facilitators/barriers	Vaccine-related transfer system	*Supply-side barriers*: no working feedback system; transfer process lacked uniformity; various approaches used by health facilities that were not systematic	N/A
India	Describing a vaccine-related problem; identifying implementation facilitators/barriers	Vaccine hesitancy; trust deficit	*Demand-side barriers*: patriarchal societal norms; social media influences; vaccine-critical views among practitioners of alternate systems of medicine *Supply-side barriers*: lack of adequate technical knowledge among frontline healthcare providers	N/A
Nigeria	Assessing or testing implementation strategies	N/A	N/A	Participatory action research approach
Nigeria	Identifying implementation facilitators/barriers; exploring potential implementation strategies	N/A	*Demand-side barriers*: male child preference; patriarchy; safety concerns; religious concerns; anti-vaccine misinformation and rumors; low perception of effectiveness/efficacy of vaccines; inaccessibility of localities; low health literacy and superstitious beliefs; traditional control mechanisms; religious beliefs *Demand-side facilitators*: institution of village head/town crier; existence of change agents; traditional control mechanisms; religion	Engagement of community service organizations
Nigeria	Identifying implementation facilitators/barriers	N/A	*Demand-side barriers*: lack of awareness/percieved benefit; role of husband and parent in-laws; competing demand occasioned by insurgency; language barriers; suspicion of vaccines	N/A
Pakistan	Describing a vaccine-related problem; identifying implementation facilitators/barriers; exploring potential implementation strategies	Vaccination in urban slums	*Demand-side barriers*: household barriers; lack of knowledge and awareness; misconceptions and fears regarding vaccines; social and cultural barriers; religious beliefs *Supply-side barriers*: underperformance of staff; inefficient funds utilization; unreliable immunization and household data; interference of polio campaigns with routine immunization	Six-step validated framework for immunization services in urban slums
Uganda	Describing a vaccine-related problem; identifying implementation facilitators/barriers	Microplanning process	*Supply-side barriers*: knowledge gaps; limited information available; bulky and complex tool; overtasked health providers	N/A
Uganda	Describing a vaccine-related problem; identifying implementation facilitators/barriers; exploring implementation strategies	District-level leadership in immunization programmes	*Supply-side barriers*: lack of participation in planning; no proper coordination	Sector-wide and intersectoral coordination of funds and activities
Vietnam	Describing a vaccine-related problem; identifying implementation facilitators/barriers	Immunization system	*Demand-side barriers*: perceptions of adverse reactions; limited knowledge *Supply-side barriers*: no legal framework; out-of-date immunization schedule; inappropriate distribution and administration of vaccines	N/A

*N/A* Information not applicable because it was not addressed by the study

The Ministry of Health and local governments were the most common audiences targeted for dissemination (Table 4) (*n* = 9; 81.8%). Meetings (*n* = 8; 72.7%) and dissemination workshops (*n* = 4; 36.4%) were the most frequent dissemination activities. Policy briefs (*n* = 11; 100%) and publications (*n* = 10; 90.9%) were the most frequently used dissemination tools. Respondents from almost all projects described perceived changes because of the project at the time of the study (*n* = 10; 90.9%) (Table 5). Respondents from more than half of  the projects described how the results of their research had been directly used at the time of the study (*n* = 6; 54.5%). The results from some projects were also used to develop tangible products to strengthen implementation (*n* = 5; 45.5%).

**Table 4:** Stakeholder engagement and dissemination

Country	Stakeholders	Dissemination activities	Dissemination products
Chad	National ministries; local government; international partners; health facilities/providers; community members	Meetings; conference	Publication; policy brief; presentation
Ethiopia	National ministries; local government; health facilities/providers; community members	Meetings; dissemination workshop; conference	Publication; policy brief; presentation; poster
Ethiopia	National ministries; local government; health facilities/providers; community members	Meetings	Publication; policy brief; presentation
India	National ministries; local government; health facilities/providers; community members	Meetings; dissemination workshop	Publication; policy brief
Nigeria	National ministries; local government; international partners; health facilities/providers; community members	Meetings, conferences, policy dialogue; media	Publication; policy brief; report; presentation; poster
Nigeria	National ministries; local government; non-governmental organizations; community members	Not described	Publication; policy brief
Nigeria	National ministries; local government; community members	Not described	Publication; policy brief; report
Pakistan	National ministries; local government; international partners; non-governmental organizations; health facilities/providers; community members	Meetings; dissemination workshops; website	Publication; policy brief
Uganda	National ministries; local government; international partners; health facilities/providers	Meetings; dissemination workshop; technical working group	Publication; policy brief; report; presentation
Uganda	National ministries; local government	Meetings; technical working group	Publication; policy brief; presentation
Vietnam	National ministries; local government; community members	Not described	Policy brief

**Table 5:** Use and perceived changes

Country	Uptake	Products	Perceived changes
Chad	Project implemented tailored communication strategy in campaign	Tailored communication strategy	Using the embedded approach in other research projects; national effort to engage nomadic communities
Ethiopia	Implemented and published tool; tool being implemented in multiple districts; capacity building project on immunization; regular supervision on immunization; developing community scorecard	Use of Community Health Data for Shared Accountability Guidelines	Conducting similar embedded implementation research on use; using tool in health service delivery as part of routine work
Ethiopia	Not described	Not described	Hospitals developed transferal slip
India	Booklet and mobile app being released for health workers	Booklet; mobile app	Religious leaders engaging in immunization activities; increase in vaccination coverage
Nigeria	Dialogues and implementation of joint action plans	Joint action plans; participatory action research tools	Increase in immunization coverage; strengthened capacity of community in participatory action research approach; continued used of participatory action research approach; participatory action research approach being applied to polio outbreak; scale-up of strategy to other areas in state; using the embedded approach in other research projects; strengthened capacity in research; better understanding of the value of evidence-informed decision-making; decision-maker engaged in further research
Nigeria	Not described	Not described	Not described
Nigeria	Not described	Not described	Agreement that immunization is an essential service for potential integration with nutrition and other services
Pakistan	Identified pockets of unimmunized children and started working to target caregivers with counseling and community mobilization	Recommendations based on immunization barriers validated by decision-makers	Appointed vaccinators to uncovered areas; coordination meeting of immunization and polio; improved microplanning and communication between health providers
Uganda	Partners used to modify microplan and conducting training; Ministry of Health developed shorter, more user friendly version of the tool; contributing to the revision of the microplanning tool	Not described	Participating in the evaluation of the microplan process; better sharing of data to complete microplan
Uganda	Not described	Not described	District recruited District Health Management Officer; more attention to areas of low performance and weakness
Vietnam	Not described	Not described	Invited to be part of further research on immunizations; increased capacity for research; using the embedded approach in other research projects

### Enablers and barriers

In this section, we present the enablers and barriers to the process of conducting the decision-maker-led research projects, as well as bringing about changes. The project process consisted of all stages from conceptualization through implementation and dissemination, while changes referred to the outcomes of the projects in terms of the use of the findings or perceived changes resulting from the projects. In each section, enablers are presented first, followed by the barriers.

#### Factors influencing the project process

##### Enabler: existing relationship or prior experience in study setting.

Almost all decision-makers and researchers knew each other prior to the study (*n* = 10; 90.9%). Some research teams also had prior experience in the study area (*n* = 4; 36.4%). Respondents described the importance of building off these existing relationships and prior experiences to facilitate their projects. According to one respondent:

“The key aspect that really made this easy is our previous relationship and also our previous partnership. […] So, I think this environment of mutual trust and also not only mutual trust but mutual learning.” (Researcher)

##### Enabler: decision-maker identified problem or country priority for research.

For some projects, the decision-maker learned about the call first, identified the problem or need to be addressed, and approached the researcher to collaborate (*n* = 5; 45.5%). Even when the researcher initiated the study, the problem or need for research was often identified by the decision-maker (*n* = 3; 27.3%). According to one respondent:

“Because then you know the programme and you analyze it routinely on a day-to-day basis and you know where the implementation challenges are. So [to] really formulate your hypothesis, which is not [a] theoretical hypothesis but […] based on the practical observations and gaps that we have identified.” (Decision-maker)

When the problem was not identified by the decision-maker or the research did not address a country priority, projects experienced challenges at later stages in engendering change (*n* = 3; 27.3%).

##### Enabler: Interest in and support for decision-maker led research.

Decision-makers from all projects had prior experience or expressed interest in engaging in research. Researchers from most projects (*n* = 9; 90.9%) also expressed support for decision-maker led research or recognized the benefits of engaging with decision-makers in research. Both decision-makers and researchers felt that this interest and support was critical to engaging in decision-maker led research and for the success of their projects. As one respondent described:

“[Decision-makers] are experts in their own field and they are experts in the...they know what government is all about. They know what will work and what will not work. So if you get into a place and you need to do some work with the government, it is better to let them really direct you. That way you are more likely to be successful.” (Researcher)

However, some researchers also expressed concern about the appropriateness of decision-maker led research for all purposes and contexts. They described the need to consider when decision-maker led research is appropriate or not before engaging in it.

“I think it depends on […] the decision-maker and their demand for evidence and their ability to use evidence, and a bit on the context. […] So I think trying to assess what is the best context in which a decision-maker may be the best lead for research is important and in situations which the researcher will be the lead but the research to be designed in a way that also uses the capacity of the decision-maker to demand and be able to use research.” (Researcher)

##### Enabler: combination of research and practice.

As conditioned in the call for proposals, decision-makers and researchers had to partner together in the project to be eligible to apply for funding. In general, researchers provided scientific and technical input to the proposal and did most of the data collection, analysis, and writing. Decision-makers, on the other hand, provided a practical and context-specific perspective that was instrumental in identifying research questions, aligning the proposal with country priorities, understanding current implementation of the immunization intervention, and facilitating the research project including engaging stakeholders and disseminating the findings. Most respondents described this combination of research and practice as positive, complementary, and contributing to the success of the project. However, a few respondents also described challenges in aligning the research project with country priorities and collaborating during the project. Respondents discussed differences between decision-makers and researchers in terms of their perspectives, approaches, priorities, and interests for engaging in research.

##### Enabler: external stakeholder engagement and dissemination throughout study.

Most projects engaged external stakeholders other than the decision-maker or researcher throughout the study period (*n* = 7; 63.6%). Some disseminated their findings during the study in addition to at the end (*n* = 6; 54.5%). Many respondents perceived this ongoing engagement and dissemination as beneficial by aligning the research project with country priorities and needs, leveraging resources for the project, improving acceptability of the findings, and identifying new opportunities to facilitate changes following the project. As one respondent described:

“Although we thought we would disseminate at the end [in] the conventional way, the dissemination, it started [a] little earlier. […] It was very common to be asked about what we have found so far. Questions about how we can improve what is happening. And sometimes in answering those questions, we were disseminating our data.” (Researcher)

For a few projects, stakeholder engagement and dissemination were planned throughout the project as a component of the research strategy (*n* = 3; 27.3%). In these studies, data collection took place in multiple phases. Each phase was followed by dissemination of the findings from the previous phase. Data collection during the next phase built on what had been found in the prior phase. This enabled studies to not only identify strategies and solutions to strengthen implementation but explore stakeholders’ perceptions about them and in some cases, test whether they worked.

##### Barrier: limited engagement of the decision-maker or researcher throughout study.

While external stakeholder engagement was identified as an enabling factor for the project, respondents from several projects also mentioned challenges regarding meaningful, ongoing engagement with their decision-maker or researcher counterparts throughout the project. Some respondents described challenges such as scheduling time to meet with the decision-maker and meaningfully engaging them in all study activities. Decision-makers described or were perceived to have competing priorities outside of the research project as part of their routine work. Some respondents also felt that decision-makers did not have the capacity to engage in certain research activities, specifically for data collection and analysis. Respondents from a few projects also discussed challenges around the meaningful engagement of the researcher. Some respondents felt that the researchers had other projects or activities that they were leading and not enough time to commit to providing adequate support necessary for the decision-maker led project.

##### Barrier: limited involvement of national-level decision-makers and international partners.

Respondents from many projects felt that they would have been more successful if they had involved more national-level decision-makers and international partners in the project. This limited involvement of national-level decision-makers was perceived to limit the scope of the changes that could be made and potential scale-up following the project. While the Ministry of Health was often a stakeholder targeted for dissemination activities, only one decision-maker serving as the Principal Investigator was affiliated with the Ministry of Health (*n* = 1; 9.1%). Respondents from this project felt the involvement of a decision-maker from the Ministry of Health was one of the main factors that contributed to their success. According to one respondent:

“So if we could have added another decision-maker, probably from the Ministry of Health, I think that dissemination and then the [uptake] of the results would have been more national rather than limited to my setting of practice.” (Decision-maker)

Other respondents described the limited involvement of international partners, specifically the country offices of WHO and UNICEF. Respondents felt that this involvement of international partners was a missed opportunity that could have been leveraged by the projects to strengthen them and support changes.

##### Barrier: insufficient capacity and resources for qualitative research methods.

All projects employed some type of qualitative research methods to generate knowledge needed to inform implementation. Respondents from some projects discussed how they were unprepared for the substantial task of undertaking qualitative research methods. Respondents discussed the need for strengthening capacities and obtaining adequate resources for qualitative research methods, specifically for the analysis and interpretation of their data. As one respondent described:

“And also another challenge was in terms of data analysis. As I said, we collected a lot of data. And analysis of qualitative data was quite difficult. Maybe that is also related to our backgrounds, as that was not our background.” (Researcher)

#### Factors influencing changes

##### Enabler: relevant and acceptable findings to inform implementation.

Respondents from many projects discussed the relevance and acceptability of the findings for informing implementation of the immunization intervention. Respondents described the research findings as useful for the decision-maker to inform implementation of the immunization intervention. They also felt that the findings were applicable in the real-world context in which they were operating. Respondents also perceived the findings to be more acceptable to other stakeholders, in part because the way the research was carried out with their ongoing engagement. This was especially important when the findings informed changes that were outside the decision-maker’s individual ability to act upon and other stakeholders were critical to their success in bringing them about. According to one respondent:

“I think one of the advantages […] was we had buy-in for lack of a better word. […]. They knew about the study and it was easy to walk to them and say […] we suspect we've found one aspect that doesn't work well, and they were more receptive of critique.” (Researcher)

##### Enabler: integrated into decision-making processes and ongoing or routine work.

Respondents from some projects discussed the integration of their project into decision-making processes and ongoing or routine work in the country. Respondents from a few projects described how the project came about at a time when they or other stakeholders were concerned about a problem and that they undertook the study to inform their decision on how it could be addressed. Respondents from one project discussed how their study was integrated into an ongoing project that enabled them to leverage networks and resources to implement changes. Respondents from another project described how the project and changes resulting from it were perceived to be part of the routine work of health systems actors. This integration was perceived to facilitate changes informed by the findings of the study. One researcher said:

“I think having a funded project in the district also working in vaccination, in line with what we were trying to understand, this was really useful. […]. So in this implementation phase they had funding for [interventions] to increase vaccination. And they literally took all […] the results from our qualitative analysis […]. They took them all, in the [intervention].” (Researcher)

##### Barrier: results did not support a clear action to improve implementation.

Respondents from several projects discussed challenges when their findings did not support a clear action to improve implementation. Some respondents felt that the focus of the projects was on understanding implementation rather than exploring or testing solutions to improve it, and this focus made changes difficult to bring about. Others felt that the findings revealed aspects or barriers that were not easy to address solely within the context of immunizations. Others felt that the changes that were needed to strengthen implementation were outside of the decision-maker’s ability to address. Some of the changes relied on decisions that needed to be made by other stakeholders that had not been engaged in the implementation research project.

##### Barrier: limited planning or support through the process of making changes.

Many respondents discussed how there was limited planning or support through the process of making changes. Respondents discussed the need for better conceptualization and planning for change from the beginning of the projects since the aim was to inform implementation. While the specific results and changes that these results might inform could not be known prior to the study, respondents felt that further thought should have been given to the type of results that were needed, how they might be used, who might need to be engaged, and what resources might be needed for these efforts before the study started. They also felt that the project did not end when the study period was over, but rather continued through a process of informing and making changes to strengthen implementation. The continuation of the projects through this process necessitated additional time and resources that were not accounted for in the planning for the study. They also described the need for commitment or resources from various partners to support the process of making changes informed by the results of the study.

##### Barrier: limited role of a single study in informing changes to strengthen implementation.

Some respondents described how one project was limited in terms of making or sustaining changes to strengthen implementation. Respondents described how the findings from this project generated new research questions that needed to be answered before a decision could be made. While this project may have been used to understand the barriers to implementation, further research might be necessary on specific barriers, the strategies that could be used to overcome them, and the impact of implementing changes. They felt that multiple rounds or phases of research or funding might be necessary to strengthen implementation, fully engage with decision-makers in research, and support the institutionalization of research into decision-making processes.

## Discussion

This study summarizes the study characteristics along with the experiences and perceptions of decision-makers and researchers across the research projects supported through the DELIR initiative, revealing several factors that influenced the process of carrying out the projects or contributed to their ability to bring about changes to strengthen the implementation of immunization interventions in different LMICs. The identification of these factors enabled us to deduce several lessons from across the projects. These lessons may be used to inform future use and design of decision-maker led strategies towards greater embedding and integration of research as a part of decision-making processes. This study builds upon prior calls and efforts to better understand the use of embedded implementation research across settings [8, 26].

Several factors were critical for the success of the projects. First, our study emphasized the importance of the research addressing a problem identified by the decision-maker or a country priority for research. Addressing these types of problems or priorities supported the relevance or usefulness of the research and has been identified as a facilitating factor for facilitating uptake of research and bringing about changes in other studies [26–29]. In addition to the decision-maker and researcher partnership, the engagement of external stakeholders throughout the study was also identified as important for the success of the projects. This ongoing stakeholder engagement and dissemination not only facilitated the process of carrying out the research projects but contributed to the acceptability of the findings. Acceptability of the findings has been shown by this study and others to contribute to research uptake and use [26–29]. We also identified the importance of engaging national-level stakeholders, specifically the Ministry of Health, as many respondents identified the limited engagement of these stakeholders as a limitation of their study.

Respondents identified the generation of results that did not support a clear action to improve implementation, limited planning and support throughout the change process, and the limited role of a single study in informing changes to strengthen implementation as barriers to bringing about changes. These barriers underscore the substantial challenge of engendering change within systems, especially when the changes supported by the research are beyond the scope of the project, the decision-maker leading the study, or  the stakeholders engaged to address. They highlight the importance of considering the research purpose and scope, the engagement and dissemination strategies to be employed, and planning for the change process from the conceptualization and design stages of the project. On the other hand, changes were facilitated when the project was integrated into decision-making processes and ongoing or routine work. This integration of research into the real-world context has been identified by others as an important consideration for this type of work [8]. These findings suggest that research done for the purpose of informing decisions and changes to strengthen implementation should be conceptualized and designed in a way that is responsive to the needs, activities, resources, and context in a particular setting.

Our findings also show that this collaborative approach to research enabled the projects to leverage existing relationships, networks, and prior experiences in country settings and bring together the perspectives of both decision-makers and researchers that contributed to their success. While this combination of research and practice was perceived by respondents to be beneficial, they also shared that collaboration was sometimes challenging or inappropriate. The issues that caused tension or conflict between decision-makers and researchers when engaging in research should be further explored and considered when planning decision-maker led strategies. Consideration should also be given to the appropriateness of the research question for decision-maker led research. Some types of questions may build upon decision-maker’s strengths in understanding implementation and context, while others may be more suited to different strategies of engaging with decision-makers or need to be carried out independently of decision-maker engagement.

The large number of proposals received in response to calls for decision-maker led research show the extent by interest of decision-makers in engaging in research. We show that this interest is important for the success of the projects. We also show that projects were more successful when decision-makers had prior experience in research. As more decision-makers have the opportunity to engage in research, their experience with research may grow and contribute to more meaningful engagement and capacity in research as well as better institutionalization of research as a part of their decision-making processes [11]. Similarly, researchers may have more opportunities to engage with decision-makers and recognize the benefits of engagement for their work. Further research is needed on the influence and impact of this engagement between decision-makers and researchers on the study and health system. This may include considerations such as how research questions evolved in response to the tacit knowledge and input of decision-makers, how the results of the research were or continue to be used to inform and strengthen implementation, whether the relationship between the decision-maker and researcher was sustained after the projects ended, and whether the decision-maker led strategy facilitated the longer term integration of research into decision-making processes.

This study has several limitations. First, the participation of more researchers than decision-makers as respondents for the interviews may have given greater emphasis to the researchers’ perspective. Second, the diversity of projects and experiences examined for this analysis made the identification of shared perspectives difficult. Some perspectives that were unique to individual projects may not be shared by others, but were still important to influencing the level of success in terms of bring about changes that those projects achieved. There may also be some social desirability and recall bias to the results as respondents may not have felt that they could openly talk or accurately recall their experience retrospectively. Given the time at which this study was carried out, i.e. shortly after some of the projects were completed, we may not have captured longer term changes resulting from the projects.

## Conclusions

Decision-maker led research is a promising strategy to facilitate the embedding of research into decision-making processes and contribute to greater use of research to strengthen implementation of proven-effective interventions, such as immunization, toward the achievement of health goals. Our analysis of the factors that influenced the process of carrying out the projects and making changes enabled us to extract several lessons from this initiative for consideration in the future design and use of the decision-maker led strategy.

## Supplementary Information

**Additional file 1. **In-depth interview guide questions

## Data Availability

The data generated and analysed during the study are included in this published article or available from the corresponding author on reasonable request.

## Abbreviations

AHPSR: Alliance for Health Policy and Systems Research
GAVI: Gavi, the Vaccine Alliance
HPSR: Health policy and systems research
LMIC: Low- and middle-income country
