# Supplementary material for: Cross-cutting lessons from the Decision-Maker Led Implementation Research initiative
Source: Health Res Policy Syst. 2021 Aug 11;19(Suppl 2):83. doi: 10.1186/s12961-021-00706-0 (PMC8356374; doi:10.1186/s12961-021-00706-0)
Supplement: Supplementary file 1 — Additional file 1. In-depth interview guide questions [file 12961_2021_706_MOESM1_ESM.pdf]

<u>In-depth Interview Guide Questions</u>

INTRODUCTION AND PROJECT DESCRIPTION

1) **First, let's begin with some information about you. Can you please give me your name, your professional title and your title on the DELIR initiative? Can you tell me a little about your background in research and any other research experience you have had in the past? Is this your first time leading a research project?**

   [Interviewer record the following in the Interview Log Sheet:]

   a) Name of Respondent
   b) Professional title
   c) DELIR title

2) **Can you briefly describe to me your project?**
   a) What problem are you solving?
   b) What are goals and objectives?
   c) What methods are you using?
   d) What were the desired outcomes from the project?
   e) What were the desired outcomes from knowledge translation and dissemination?
   f) Can you briefly describe to me the context in which your project exists: what level are you working in, who are the key stakeholders; and what are the local political and economic factors that could influence your project?

PRE-SUBMISSION PHASE

3) **Please describe to me how the project came about before the submission to AHSPR?**
   a) Who came up with the idea? How did you find out about the call for proposals?
   b) How was the decision made to conduct this project and submit a proposal to AHSPR?
   c) Who wrote the first draft of the proposal for submission to AHSPR?
   d) How did you define roles at this stage? Who was responsible for what task? Were these defined beforehand?
   e) How long did it take?
   f) What worked well and didn't work well at this time?

4) **I would like to ask about the <u>decision-maker-researcher relationship</u>.**
   a) How was the co-PI selected? Did you know each other prior to this time?
   b) Do you have a history of working together? Please elaborate.
   c) Can you describe to me how you communicated throughout the process? How often did you communicate?
   d) How did you define your roles and working relationship?
   e) What do you think worked well and didn't work well about this format?
   f) How did you resolve any obstacles in the implementation of the project?
   g) Do you have plans to work together in the future? Please elaborate.

SUBMISSION AND PROTOCOL DEVELOPMENT PHASE

5) **I would like to discuss with you the time right after your protocol was accepted by AHSPR. Can you describe to me your experience at the Protocol Development Workshop and the time up to the protocol was completed?**
   a) Who was involved at this stage of the project
   b) What challenges did you face at this stage?
   c) How were the challenges addressed?
   d) Who was involved in making decisions at this stage?
   e) What is the process of making decisions at this stage?
   f) Where there any procedures in place for interacting with the research team? Can you describe to me what these procedures were?

6) **Can you list for me the <u>partners</u> on the project (other than the PI and researcher)? How were these partners and collaborators identified?**
   a) Who agreed to be part of the project? Was this through a formal or informal partnership?
      i) Specifically probe about:
         (1) Government organizations: research institutions; advisory bodies; committees
         (2) Government-supported organizations: Think tanks; technical agencies; academia; consultants
         (3) Independent organizations: NGOs; bi-laterals; multi-laterals; consortia; academia
   b) Who from this group would you say are the active partners?
      i) Why do you think they are key partners? Do they provide funding; time; information?
      ii) Can you tell me what you think about quality of your relationship with these partners?
      iii) What do you think is the level of knowledge of research methods and terminology among partners?

IMPLEMENTATION PHASE

7) **I would like now to ask you about the <u>implementation phase</u> of the project. Can you tell me the steps followed during the implementation stage?**
   a) Who was involved at this stage of the project
   b) What challenges did you face at this stage?
   c) How were the challenges addressed?
   d) Who was involved in making decisions at this stage?
   e) What is the process of making decisions at this stage?

DISSEMINATION PHASE

8) **I would like to now ask you about how results and findings from the study were shared and communicated. Can you tell me what the dissemination plan was and what were your objectives for this stage?**
   a) What was the mode of interaction with partners at this stage?
   b) Where you able to disseminate the results in the way you had planned? Why or why not?

c) What activities – written, oral, formal or informal – were done to introduce the evidence to partners and decision-maker?
d) What was the response the findings? Was the response as anticipated? Why or why not?

9) What are the next steps in the project? How will the results and findings be used beyond the communication and reporting plan agreed to with AHSPR?

a) Have the results been used by you or others in any way during or since the conclusion of the project?
b) Have the findings changed or influenced how things are being done at the community level or by decision-makers? Please elaborate.
c) Are there any tools, instruments or activities that have emerged from the findings of this project? Please elaborate.

10) I would like to now know more about <u>your experience</u> working on this project

a) What did you find worked well and did not work well in relation to how this embedded research led to improvements and action?
b) What did you find worked well and did not work well in relation to conducting research, and the effectiveness of embedding research methods and approaches in the process?
c) What was your experience working with AHSPR? What worked well or didn't work well? What could they have done differently to support or improve your project?
d) What would you do differently if you had to do this project again?

11) Is there anything that we didn't discuss that you would like to add?

Thank you for your willingness to participate. If you have any questions about the interview or this study, please feel to contact me. We hope that you will be willing for us to follow up with any questions. We appreciate your time and valuable input.

End of interview. [Record end time on Interview Log Sheet]
